# Reward-based behaviors and emotional processing in human with narcolepsy-cataplexy

**DOI:** 10.3389/fnbeh.2013.00050

**Published:** 2013-05-23

**Authors:** Sophie Bayard, Yves A. Dauvilliers

**Affiliations:** Department of Neurology, National Reference Network for Narcolepsy, Gui-de-Chauliac Hospital, CHU Montpellier, INSERM U1061, University of Montpellier 1Montpellier, France

**Keywords:** hypocretin, orexin, cataplexy, narcolepsy, mood, reward, decision-making, addiction

## Abstract

Major advances in the past decade have led a better understanding of the pathophysiology of narcolepsy with cataplexy (NC) caused by the early loss of hypothalamic hypocretin neurons. Although a role for hypocretin in the regulation of sleep/wakefulness state is widely recognized, other functions, not necessarily related to arousal, have been identified. Hence, the hypocretin system enhances signaling in the mesolimbic pathways regulating reward processing, emotion and mood regulation, and addiction. Although studies on hypocretin-deficient mice have shown that hypocretin plays an essential role in reward-seeking, depression-like behavior and addiction, results in human narcolepsy remained subject to debate. Most of studies revealed that hypocretin-deficient narcolepsy patients either drug-free or medicated with psychostimulant had preferences toward risky choices in a decision-making task under ambiguity together with higher frequency of depressive symptoms and binge eating disorder compared to controls. However, human studies mostly reported the lack of association with pathological impulsivity and gambling, and substance and alcohol abuse in the context of narcolepsy-cataplexy. Prospective larger studies are required to confirm these findings in drug-free and medicated patients with narcolepsy. Inclusion of patients with other central hypersomnias without hypocretin deficiency will provide answer to the major question of the role of the hypocretin system in reward-based behaviors and emotional processing in humans.

## Introduction

The orexins (orexin A and B) (Sakurai et al., 1998) or hypocretins (hypocretin 1 and 2) (De Lecea et al., 1998) are neuropeptides produced by several thousand neurons restricted to the lateral hypothalamus (Peyron et al., 1998; Date et al., 1999; Nambu et al., 1999). These neurons project widely to the olfactory bulb, cerebral cortex, thalamus, hypothalamus and brainstem, and particularly densely to the locus coeruleus, tuberomamillary nucleus, raphe nucleus, and bulbar reticular formation (Peyron et al., 1998; Date et al., 1999; Nambu et al., 1999). The actions of hypocretins are mediated via two orphan G protein-coupled receptors, orexin-1 (OX_1_R) and orexine-2 (OX_2_R) receptors (also known as Hcrtr-1 and Hcrtr2). In the central nervous system, Hcrtr-1 and Hcrtr2 mRNA have different and complementary expression patterns suggesting that these receptors have distinct physiological roles through different neuronal pathways (Marcus et al., 2001). Hcrtr-1 mRNA was observed in the limbic cortex but also detected in the hippocampus and in paraventricular thalamic nucleus, ventromedial hypothalamic nucleus, dorsal raphe nucleus, and locus coeruleus. In contrast, Hcrtr2 mRNA was found more diffusely in the cortex and more prominent in septal nuclei, hippocampus, medial thalamic groups, raphe nuclei, paraventricular nucleus, and ventral premammillary nucleus.

Studies in basic neuroscience have largely documented the role of hypocretin-1 and -2 in a variety of physiological processes such as arousal and the maintenance of waking (Sutcliffe and De Lecea, 2002; Taheri et al., 2002; Adamantidis et al., 2007) feeding behavior and energy metabolism (Willie et al., 2001), neuroendocrine and autonomic function and regulation of cardiovascular function (Samson et al., 2005). Subsequently, studies of efferent and afferent projections of hypocretin neurons revealed the implication of the hypocretin system in the mesolimbic pathways regulating emotion, reward processing and addiction (Boutrel et al., 2005; Harris et al., 2005).

Narcolepsy with cataplexy (NC) is a rare debilitating neurological disorder that affects approximately 0.026% of the general population. The disease is characterized by excessive daytime sleepiness (EDS), cataplexy (i.e., sudden weakening of muscle tone triggered by emotions), fragmented nocturnal sleep, and other dissociated manifestations of rapid eye movement sleep phenomena such as sleep paralysis and hypnagogic hallucinations (Dauvilliers et al., 2007). In addition, to sleep symptoms, NC is also associated with reduced quality of life (Ervik et al., 2006; Dodel et al., 2007; Dauvilliers et al., 2009; Vignatelli et al., 2011), mood symptoms (Dauvilliers et al., 2009; Fortuyn et al., 2011) and cognitive alterations (Bayard et al., 2012a). NC is caused by an extensive loss of hypothalamic neurons producing hypocretin (Peyron et al., 2000; Thannickal et al., 2000). Accordingly, low/undetectable cerebrospinal hypocretin-1 is observed in almost all patients with NC (Dauvilliers et al., 2007). The specific physiopathology of NC provides a fascinating insight into the roles of hypocretin in human behavioral regulation.

In this review, we describe and discuss physiological, behavioral and neuroimaging data from human NC that favors the role of the hypocretin system in reward-based behaviors, emotion regulation, decision-making processing, mood symptoms, and addiction.

## Regulation of satiety

Food intake is partially mediated through brain pathways for motivation and reinforcement with close interaction with the reward processing (Kelley et al., 2005; Wise, 2006). The question of whether common brain mechanisms mediate the acquisition and development of eating and addiction habits remains unknown. Food is a daily necessity and obesity is largely attributed to excess caloric intake induced by excessive hedonic drive even in condition with normal metabolic need (Wise, 2013). In some cases, obesity may reflect addiction to high-energy foods with parallels to addiction behavior toward drug and alcohol. Recent advances have been made in identifying the metabolic feedback signals and neural systems, located mainly in brainstem and hypothalamus that represent a “homeostatic” energy balance regulatory system (“wanting” for food), but also the neural pathways and functions mainly located in cortico-limbic structures referred to as “hedonic” drive of eating (“liking for food”) (Berridge et al., 2010; Berthoud, 2011). Limbic areas such as nucleus accumbens and ventral pallidum play a central role in the “liking” food intake. The latter system referred to external sensory information processing, reward processing, and cognition and executive functions where opioid, endocannabinoid and orexin signals modulate the sensory pleasure. Midbrain dopamine neurons with projections to the nucleus accumbens in the ventral striatum have been strongly implicated in food and drug reward with the potential to strongly affect the amount of energy consumed (Kalivas, 1993; Wise, 2006, 2013; D'Ardenne et al., 2008). Dysfunction in reward circuits may thus contribute to obesity and eating disorders with neurochemical evidence (i.e., alteration in dopamine or opioids) of abnormal reward-related brain functions in animal models of binge eating, bulimia, and anorexia nervosa (Berthoud et al., 2011; Avena and Bocarsly, 2012).

The recent discovery of orexin/hypocretin and melanin concentrating hormone expressing neurons in the lateral hypothalamus acted as metabolic sensors and modulated energy homeostasis, hedonic pleasure, decision-making to make optimal adaptive choices, and reward-seeking (Yoshida et al., 2006; Berridge et al., 2010). A close interaction between the hypocretinergic and dopaminergic systems in satiety control was also supported by data showing that orexin in the posterior ventral pallidum can enhance liking for sweets rewards as with mu opioid stimulation (Ho and Berridge, 2013). NC is associated with excessive weight gain particularly at disease onset and in children (Okun et al., 2002) with a global increased frequency of obesity also in adults (Dahmen et al., 2001). Interestingly, weight gain in NC appears not related to EDS (i.e., inactivity) (Kok et al., 2003) or higher food intake from controlled diary study (Lammers et al., 1996). As hypocretin was primarily known to regulate feeding behavior and metabolism (Sakurai, 2003), some studies were recently conducted to explore eating disorders prevalence in NC.

In a small clinical sample, Chabas and colleagues reported that patients with typical cataplexy (*n* = 7) had higher scores on the Eating Attitude Test (EAT-40) and had more frequent features of bulimia nervosa compared to controls (*n* = 9) (Chabas et al., 2007). Fortuyn and colleagues showed that patients with NC display various symptoms of eating disorders. In their case-controlled study, 23.3% of the patients fulfilled the criteria for a clinical eating disorder, and none in controls (Fortuyn et al., 2008). Four patients (6.7%) had bulimia nervosa, only one had anorexia nervosa, and nine (15%) were classified as Eating Disorder-Not Otherwise Specified (i.e., subthreshold of bulimia nervosa, binge eating disorder). On the symptom level, 25% of patients reported binging twice a week or more often. Interestingly, these results were essentially the same when patients were compared to BMI-matched controls and were note related to medication use. Same results were obtained by Dimitrova and colleagues who reported higher scores on the Binge Eating Scale in patients with NC, with moderate or severe binge eating in 23% (Dimitrova et al., 2011). Finally, a recent study aimed at assessing the prevalence of sleep-related disorder (SRED) in NC (Palaia et al., 2011). SRED is a non-rapid eye movement parasomnia defined by recurrent episodes of involuntary eating and drinking during arousals from the main sleep period. In this cohort of 65 consecutive adult patients with NC, SRED was significantly associated with NC (32 vs. 3% in BMI-matched controls). Interestingly, SRED in NC was not associated with weight gain suggesting a possible primary effect of hypocretin deficiency. Finally, by examining 166 patients with NC and 80 controls with self-report structured interview, no difference was found in the current prevalence of eating disorders (i.e., bulimia nervosa, binge eating disorder, or anorexia nervosa), nor was the frequency of eating attacks higher in the narcolepsy group (Dahmen et al., 2008).

In conclusion, even being still controversial, patients with NC may present an increased frequency of subtle changes in eating behavior with binge eating and compulsive sleep related-eating disorder. However, eating disorders *per se* could not explain the positive energy balance leading to frequent overweight in patients with narcolepsy-cataplexy. In the future, it would be worthwhile to use more precise instruments including those measuring satiety after standardized meals and to measure basal metabolic rates by indirect calorimetry. It remains also required to study the brain reward mechanisms that generate homeostatic energy balance regulatory and hedonic drive of eating systems and their interactions in hypocretin-deficient patients with narcolepsy-cataplexy.

## Emotional processing

Several models of emotion highlight the role of particular brain regions in emotion identification, response and regulation that involve cortical, subcortical and limbic (i.e., amygdala and medial prefrontal cortex) structures. The amygdala plays a major role in interpretation of emotionally significant stimuli, with strong projections to the hypocretin area (Sakurai, 2005). Recent studies have explored the structural and functional integrity of the amygdala in human NC. One study using magnetic resonance imaging (MRI) spectroscopy suggested amygdala involvement in NC (Poryazova et al., 2009). As in patients with amygdala lesions, patients with NC failed to exhibit startle potentiation during unpleasant stimuli (Khatami et al., 2007). A psychophysiological investigation in NC also revealed an attenuated reaction to unpleasant pictures (Tucci et al., 2003). Recent functional MRI studies examined brain activation patterns in patients with NC when shown humorous material. One of these studies revealed an enhanced ventral striatum and hypothalamus response in patients with NC while shown humorous cartoons (Reiss et al., 2008). In contrast, positive humorous pictures elicited reduced hypothalamic response together with pronounced activity in amygdala in another NC study (Schwartz et al., 2008). Recently, the same group reported a reduced amygdala activity during aversive conditioning together with an increased activity in the bilateral amygdala and the dorsal striatum during positive emotional stimuli in NC (Ponz et al., 2010a,b). All these findings suggest an amygdala involvement in the pathophysiology of NC, which would result in abnormal emotional processing under both pleasant and unpleasant conditions. Very recently, we have explored a classical emotional processing (i.e., facial affect recognition) in patients with NC compared with patients with central hypersomnia without cataplexy and healthy controls (Bayard et al., 2012b). As most of previous studies suggested the involvement of amygdala structure in the perception and judgment of fear (Adolphs et al., 1994; Morris et al., 1996) as well as happiness and sadness (Fusar-Poli et al., 2009), we hypothesized that patients with NC would show impaired recognition of these emotions in facial expressions. Furthermore, we were also interested to study their emotional regulation strategies (using a self-rated questionnaire) based on the frequent coping behavior in NC to avoid specific emotional situations that trigger cataplexy. We found that patients with NC did not significantly differ from controls in their emotional judgment (valence and arousal) with normal emotion recognition in facial expressions as well as in their explicit emotional regulation strategies.

In conclusion, neuroimaging studies provided convincing evidence for a central role of the amygdala in the pathophysiology of human NC. Observation of both exaggerated and reduced amygdala activation remains puzzling. This observation might probably reflect the complex interaction and synergy between amygdala and other limbic brain regions (i.e., prefrontal cortex, nucleus accumbens, ventral striatum) depending on the nature of the processing elicited by the stimuli used (i.e., reward, aversive conditioning or humorous pictures processing). Finally, the normal performance on facial expression recognition observed in NC may rely on the recruitment of alternate brain circuitries that exclude the amygdala pathway. An alternate hypothesis is that the metabolic changes inconstantly reported in the amygdala structure in NC (Poryazova et al., 2009) are not sufficient to alter the emotion recognition signaled by facial expressions. An overlap between the emotional and the social brain may thus be emphasized with promising perspective to identify new pathophysiological pathways in studying emotion in hypocretin-deficient patients with narcolepsy.

## Decision-making processing

Hypocretin system is implicated in reward processing and addiction by sending direct and indirect projections to dopaminergic regions i.e., the ventral tegmental area, the nucleus accumbens, and the amygdala (Sakurai, 2007; Aston-Jones et al., 2009). Given that subjects with altered feedback sensitivity showed decision-making problems, and that impulsive decision-making was a condition of intolerance to delay-of-reward, several studies were interested in assessing decision-making and impulsivity in pathologies associated with dopamine system abnormalities or dopaminergic medication. Hence, poor decision-making performances and impulsivity were reported in psychiatric (e.g., drug dependence and pathological gambling) and neurological pathologies (Parkinson's disease and restless legs syndrome), all disorders being characterized by an abnormal reward-mediated processing involving the dopaminergic system (Dunn et al., 2006; Bayard et al., 2010; Kertzman et al., 2011; Fond et al., 2012).

In neuropsychological research, decision-making is commonly assessed with laboratory tasks such as the Iowa gambling task (IGT) (Bechara et al., 2000), the game of dice task (GDT) (Brand et al., 2005), or the Balloon Analog Risk Task (BART) (Lejuez et al., 2002). The IGT is based on reinforcement learning, which measures the ability to balance short-term rewards against long-term losses where premises, outcomes, rewards, or punishments are initially uncertain (decision under ambiguity). In contrast, in the GDT, the potential consequences of different options and their subsequent probabilities rely on explicit information (decision under risk). The BART involves a variable number of choices in a context of increasing risk. We were the first to report reduced performance on decision-making under ambiguity (IGT) in drug-free patients with NC in contrast to normal decision-making under explicit conditions (GDT) (Bayard et al., 2011a). In our work, patients with NC made disadvantageous choices involving immediate high reward (monetary gain) whereas future high punishment (monetary loss) had little influence. Our results were further confirmed in a modest sample size of drug-naïve and medicated patients with NC without statistical power to compare patients taking or not stimulants (Delazer et al., 2011).

Most of psychostimulants (modafinil, methylphenidate, and amphetamines) used to manage sleepiness in NC increase wakefulness potentially through inhibition of the dopamine transporter and augmentation of cortical and caudate dopamine concentrations (Wisor and Eriksson, 2005). Recently, two case reports described the association between modafinil, pathological gambling, and severe mania in the context of NC (Tarrant et al., 2010; Crosby et al., 2011). Given that background, we were interested in comparing addiction, pathological gambling and risk-taking on the IGT and the GDT in drug-free, stimulant-medicated patients with NC, and controls. We demonstrated on a large clinical sample that patients with NC either drug-free or medicated with psychostimulant selected more disadvantageous choices under ambiguous conditions (IGT) than controls, without differences between patients treated or not (Bayard et al., in press). Furthermore, we found that psychostimulants did not increase risky-taking behavior on the GDT, substance addiction and pathological gambling occurrence. In addition to the lack of relationship between the IGT performances and many characteristics of NC, our recent results favor a direct impact of the hypocretin deficiency in triggering the risky-taking behaviors in NC. Finally, another study using the BART reported normal risk-taking behavior in patients with NC or with narcolepsy without cataplexy, but patients with cataplexy were more impulsive and more prone to binge eating (Delazer et al., 2011; Dimitrova et al., 2011).

Impulsivity and sensation-seeking are two behavioral dimensions being closely related to the concepts of decision-making and rewarding (Zermatten et al., 2005; Bayard et al., 2011b; Dretsch and Tipples, 2011). To the best of our knowledge, only few studies focused on self-reported impulsivity and sensation-seeking behaviors in NC but with non-robust results. We have explored the four facets of impulsivity proposed by Whiteside and Lynam (2001) using the UPPS Impulsive Behavior Scale in patients with NC. The first dimension of the scale, urgency, refers to difficulty resisting strong impulses driven by negative affect. The second dimension, premeditation, is characterized by an inability to consider the potential consequences of one's behavior. The third dimension, perseverance, refers to the lack of ability to stay on task while experiencing boredom. Finally, sensation-seeking refers to an individual's need for excitement and stimulation. In a first study, we found that drug-naïve patients with NC showed lack of perseverance on the UPPS (Bayard et al., 2011a). However, on a larger sample of drug-free and medicated patients matched to healthy controls, we were unable to reproduce this observation (Bayard et al., in press). Regarding the discrepancies observed on the UPPS, sensitivity analyses indicated no significant difference between patient's characteristics from our first study (Bayard et al., 2011a) compared to those from our second study (Bayard et al., in press). Discrepancy between both studies may be due to type I error risk due to the small population of subjects included in the previous study. On the Eysenck Impulsiveness Scale, Dimitrova and colleagues found that patients with NC scored higher than controls (10 vs. 0%), while there was a trend for the patients with narcolepsy without cataplexy (6.7%) to score higher than controls (Dimitrova et al., 2011). In addition, similar scores on the five dimensions of the Zuckerman Sensation Scale (i.e., general sensation seeking, thrill and adventure seeking, experience seeking, disinhibition, and boredom susceptibility) were found among patients with narcolepsy with and without cataplexy and controls.

In conclusion, patients with NC either drug-free or medicated with psychostimulant had reduced performances in decision-making under ambiguous condition compared to controls, without differences between patients treated or not. The performance discrepancies found between decision-making under ambiguous (IGT) but not risky conditions (GDT, BART) may be linked to different cerebral networks involved under these conditions. In fact, brain lesion and neuroimaging studies have confirmed that the fronto-cortico-striatal loop is crucially involved in decision-making under risk, whereas the limbic loop (i.e., orbitofrontal/ventromedial prefrontal cortex and amygdala) is preferentially involved in reward processing under ambiguity (Brand et al., 2007). As hypocretin neurons densely project to these limbic reward-associated brain regions, we may suggest that hypocretin deficiency in NC trigger abnormal performances in decision-making under ambiguity. Finally, there is no robust evidence for any clinically-significant association between impulsivity and hypocretin-deficient patients with NC.

## Mood symptoms

Hypocretins act primarily as excitatory neurotransmitters to control monoaminergic and cholinergic neuron activities. Hypocretin deficiency induces a cholinergic–monoaminergic imbalance, with primary effect on vigilance as well as other functions including mood regulation. As hypocretins are also involved in neuroendocrine functions and stress reactions through stimulation of the hypothalamus–pituitary–adrenal axis (Kuru et al., 2000), hypocretin deficiency *per se* may trigger mood disturbances and psychological alterations through diverse pathways.

One recent animal study supported the specific role of hypocretin receptors in the modulation of depression-like behavior. After genetic or pharmacologic inhibition of hypocretin receptor signaling, the hypocretin activity balance at either Hcrtr1 or Hcrtr2 produced an antidepressant- or prodepressant-like effect depending on the subtype activated (Scott et al., 2011). Hcrtr1 null mice displayed a significant reduction in time immobile and in latency to first bouts of immobility in the forced swim test, consistent with reduced depression-like behavior. Pharmacologic inhibition of hcrtr1 also produced the same pattern of behavior, but without any impact on anxiety level as shown by the results on the elevated plus maze and the light-dark box. In contrast, hcrtr2-null mice displayed an increase in behavioral despair but again without any effect on measures of anxiety.

High levels of psychopathology were frequently reported in cross-sectional studies in narcolepsy, with high rate (from 15 to 37%) of moderate to severe self-reported depressive symptoms (Roy, 1976; Kales et al., 1982; Krishnan et al., 1984; Mosko et al., 1989; Vandeputte and De Weerd, 2003; Vignatelli et al., 2004, 2011; Dodel et al., 2007; Dauvilliers et al., 2009; Bayard et al., 2011a, 2012a). A case-control study found mood disorder symptoms in one-third of narcolepsy patients, but with similar frequency of formal mood disorder diagnoses (Fortuyn et al., 2010). In contrast over half the patients had anxiety or panic attacks, and 35% had anxiety disorders. Interestingly, in a 5-year cohort study of patients with narcolepsy-cataplexy, mood symptoms remained relatively stable, with 25% of patients showing constant moderate to severe mood symptoms across assessments (Vignatelli et al., 2011). Our large cross-sectional narcolepsy study found that depressive symptoms (using the BDI-II) were associated with greater EDS severity, greater alterations in physical and mental health quality of life, as well as increased frequency of REM sleep manifestations such as cataplexy, hypnagogic hallucinations, and sleep paralysis (Dauvilliers et al., 2009). In addition, we found that anticataplectics at doses prescribed for cataplexy management were ineffective in treating depressive symptoms.

In conclusion, a high frequency of depressive symptoms has been systematically reported in NC, supporting the hypothesis of an endogenous mood problem dysregulation. Nevertheless, a “subsyndromal form of depression” would be more appropriate to qualify the mood disorder in NC.

## Addiction

Drug addiction is a relapsing disorder characterized by compulsion to seek and take drugs with major link to dysregulation of brain regions that mediate reward and stress. Animal studies have undoubtedly documented the role of the hypocretinergic system in addiction (Aston-Jones et al., 2009). Hypocretin neuron activities varied in proportion to drug or food preference and their neurotransmission is needed for reinstatement of an extinguished preference for morphine (Harris et al., 2007) and cocaine-seeking (Boutrel et al., 2005). Do these data from experiments conducted in laboratory animals have any correlates in humans? In seminal review papers on this topic, it is currently stated that “… *human narcoleptics, who have few or no orexin neurons, rarely exhibit stimulant abuse and seeking despite the fact that they are treated for years with stimulants* …” (Aston-Jones et al., 2009) or “… *patients with narcolepsy are often treated using addictive amphetamine-like drugs such as methylphenidate, amphetamine and γ-hydroxybutyrate, but rarely become addicted to these drugs* …” (Tsujino and Sakurai, 2009). Nevertheless, to the best of our knowledge, no data in humans have validated this clinical observation stating that hypocretin deficiency constitutes a protective factor against the development of addiction. One isolated study performed on a modest sample size population documented that substance and alcohol abuse and compulsive gambling were similar between patients with NC, patients with narcolepsy without cataplexy and healthy controls (Dimitrova et al., 2011). Our clinical experience revealed that patients with NC easily stop their psychostimulant medication (i.e., due to adverse effects, during pregnancy or for research purposes) without frequent occurrence of withdrawal symptoms in contrast to the frequent rebound of cataplexy after stopping antidepressant anticataplectic medication. However, similar observations were noted when stopping psychostimulant in other central hypersomnia conditions characterized by normal hypocretin activity (i.e., idiopathic hypersomnia with and without long sleep time, and 80% of narcolepsy without cataplexy). Although the behavioral pharmacological profile of methylphenidate, cocaine and d-amphetamine shared some similarities, the actual rates of abuse in animals and in humans are much lower for methylphenidate (Kollins et al., 2001). Altogether, we cannot conclude that the amphetamine-like drugs used to manage sleepiness in NC have particular addictive properties.

In conclusion, even if hypocretin deficiency that characterized the physiopathology of NC provides a fascinating insight into the roles of the hypocretins in addiction development, literature is lacking of robust data to document this topic in human narcolepsy. Understanding the function of basic brain stress and reward systems in hypocretin-deficient patients with narcolepsy may provide novel targets for treatment and prevention of addiction.

## Conclusion and perspectives

The specific physiopathology of NC defined by hypocretin deficiency provides a fascinating insight into the roles of hypocretin in human behavioral regulation. Although a role for hypocretin in the regulation of sleep/wakefulness state is widely recognized, other functions, not necessarily related to arousal, have been identified. Animal studies have shown that hypocretin plays an essential role in reward-seeking and addiction; however, patients with narcolepsy-cataplexy presented risky choices in a decision-making task under ambiguity together with higher frequency of depressive symptoms and binge eating disorder but without any significant association with pathological impulsivity and gambling, and substance and alcohol abuse.

Prospective larger studies are required to confirm these findings in narcolepsy but also in the context of other central hypersomnias without hypocretin deficiency to answer the question of the role of the hypocretin system in reward-based behaviors and emotional processing in humans. Further interventional studies are also needed to explore whether management of sleepiness using psychostimulant modified mood symptoms, gambling, risky behaviors and addiction in central hypersomnia disorders. Finally, the future use of non-selective hypocretin-1 and -2 agonists and antagonists appears particularly promising to treat several neuropsychiatric disorders, including narcolepsy, insomnia, but also drug addiction.

### Conflict of interest statement

This was not an industry-supported study. Yves A. Dauvilliers has received funds for speaking and board engagements with UCB, Cephalon, Jazz, Novartis, and Bioprojet. The other authors declare that the research was conducted in the absence of any commercial or financial relationships that could be construed as a potential conflict of interest.
